# The association between weight loss and engagement with a web-based food and exercise diary in a commercial weight loss programme: a retrospective analysis

**DOI:** 10.1186/1479-5868-8-83

**Published:** 2011-08-02

**Authors:** Fiona Johnson, Jane Wardle

**Affiliations:** 1 Cancer Research UK Health Behaviour Research Centre, Department of Epidemiology and Public Health, University College London, Gower Street, London, WC1E 6BT, UK

## Abstract

**Background:**

The Internet provides a widely accessible platform for weight loss interventions. Automated tools can allow self-guided monitoring of food intake and other target behaviours that are established correlates of weight change. Many programmes also offer social support from the virtual community. The aim of this research was to assess associations between engagement with self-monitoring tools and social support, and weight loss in an online weight-control programme.

**Methods:**

This paper describes a retrospective analysis of weight change among 3621 subscribers to a commercial Internet-based weight loss programme. Participants were all subscribers (2979 women; 642 men) joining the programme between July 2005 and November 2008 with two or more recorded weights spanning at least 28 days of participation in the programme. Engagement was indexed with frequency of using online diet and exercise diaries and with use of the social support forums.

**Results:**

Programme engagement was associated with weight loss in both men and women after controlling for initial BMI and duration of participation. The three engagement variables accounted for 13% of variance in percentage weight loss in women (p < .001) and 19% in men (p < .001). In analyses including all the engagement variables, exercise diary use was an independent predictor of weight loss among men, but non-significant in women. In contrast, use of the online forums was associated with weight loss in women but not in men. Among participants who were overweight or obese, those in the highest tertile of engagement with food diaries (vs the lowest) were more likely to achieve clinically significant (> 5%) weight loss (men: OR = 3.45 p < .001; women: OR = 5.05 p < .001). Being in the highest tertile of engagement with exercise diaries was associated with clinically significant weight loss in men (OR = 3.48 p < .001) and, less strongly, in women (OR = 1.46 p < .05).

**Conclusions:**

Use of self-monitoring tools and participation in online support are predictive of weight loss in the context of a commercial, online weight control programme.

## Introduction

The desire to lose weight is widespread in affluent western countries and efforts to achieve a neutral or negative energy balance are appropriate both for overweight/obese adults who are trying to lose weight and those of normal weight who wish to avoid weight gain [1,2]. Alongside services offered by healthcare providers that are typically directed towards more severely overweight groups, a wide array of commercial weight loss programmes is available [3]. People seeking assistance with weight control can choose from programmes using a variety of formats including books, DVDs, Internet sites and weight loss clubs, and offering a range of tools, services and types of support. This makes it possible for consumers to match their preferred approach to weight control to the service of a particular provider. Research into the mechanisms of operation and effectiveness of these commercial programmes has been sporadic and limited, with many popular approaches used by large numbers of customers worldwide having had little scientific evaluation. However, where research has been carried out, the results have been broadly positive, indicating that commercial programmes can be successful in assisting with weight control [4-6] while meeting users' nutritional needs [7,8].

As online facilities have become more accessible, there has been increasing use of the Internet as a platform for both commercial and health service weight loss programmes [9]. Internet programmes have been shown to achieve weight loss outcomes comparable to face-to-face interventions that use similar tools [10-12]. The potential benefits of online interventions include automatic monitoring of usage patterns among participants [13]. They may also provide considerable savings of time and cost [9]; in particular reducing demands on health professionals and therapists. Programmes typically include a variety of features, and allow participants to be selective about the tools they use. Several studies have examined which features of Internet weight loss programmes are associated with successful weight loss in the context of randomised controlled trials (RCTs) or formal research studies [9,13,14], but a recent systematic review concluded that not enough was known about components of web-based interventions associated with effectiveness [15]. It has also been suggested that patterns of use and outcomes differ between RCT participants and non-trial users of web-based programmes [16], and although many people seeking to lose weight do so without the formal setting of a therapeutic intervention or clinical trial, few studies have examined utilisation patterns and weight loss in self-guided 'direct-to-consumer' commercial services [17,18].

Two features of internet weight loss programmes that have been associated with weight loss are use of self monitoring tools and peer social support. Self-monitoring of weight and target behaviours has long been recognized as an important tool for weight control [19,20], and keeping diary records of diet, exercise or weight is associated with successful weight loss and weight maintenance [14,20-22]. Reports from programme participants also support the value of self-monitoring [23]. It appears to be the act of self-monitoring rather than the exact approach that is valuable for weight control, since there is little effect of recording method, for example electronic versus paper diaries [24], the degree of detail in the records kept [25], or whether or not participants receive training in recording accuracy [26]. Peer social support is similarly perceived to be valuable by many of those using internet weight loss programmes and may enhance outcomes and commitment to the programme [14,27-29], although active involvement in peer chatrooms and message forums has been reported to be low [17].

In this study we examined associations between several aspects of programme engagement and weight loss in 3621 people enrolled in Nutracheck: a direct-to-consumer, Internet weight loss programme centred around self-monitoring of diet and physical activity and provision of social support through an online message forum. The analysis tests the hypothesis that greater engagement either with self-monitoring tools or the social support forum will be associated with greater weight loss among all participants and clinically significant weight loss in overweight and obese participants. Men and women were analysed separately since other studies have found gender differences in use of online facilities and factors associated with weight loss success (e.g. [30,31]).

## Methods

### The programme

Nutracheck provides a platform for completing food and exercise diaries online, in return for a monthly subscription charge. The service is a self-help tool assisting the user in recording diet and exercise and charting their progress towards a target weight. The user registers online and enters personal information about height, weight, activity levels, and desired amount and speed of weight loss. The system then sets a personal daily calorie target (Harris-Benedict equation [32]) adjusted for activity level [33] and the individual's chosen rate of weight loss up to a maximum of 2l bs (0.9 kg) a week. The user is advised to use a daily food diary which links to a database of over 40000 branded and unbranded food items, automatically calculating an estimate of calorie intake. A daily exercise diary encourages additional physical activity by setting a target to expend a minimum of an extra 200 calories/day, and calculates an estimate of expenditure from the activity records. The core concept of this web-based programme is that visually demonstrating energy balance helps the user learn how to adjust their diet and lifestyle to lose weight. The website also includes tools to support behaviour change such as weight charting software, access to health and nutritional information, and an active online social community to provide support and motivation.

### Inclusion criteria

A retrospective analysis was carried out based on self-reported weight loss data from participants joining the programme between July 2005 and November 2008 who met inclusion criteria of: i) paying at least one month's subscription to the site and ii) recording two or more weights spanning a period of at least 28 days (to exclude those whose participation in the programme had been too short to give reliable weight change data). All subscribers had given their permission for use of anonymised data when signing up with the programme.

### Measures

*Percentage weight loss*. This was calculated using the first and last weights recorded on the website and was the primary outcome variable. The distribution of this variable showed moderate departure from normality (men: skewness = 0.7 [SE = 0.1], kurtosis = 2.2 [SE = 0.2]; women: skewness = 1.0 [SE = 0.5], kurtosis = 2.8 [SE = 0.9]). Exploratory analyses carried out using a square root transformation on the dependent variable showed similar results and significance levels to those using the untransformed variable, and therefore untransformed data was used in the analyses presented here to facilitate interpretation. Duration of programme use. This was calculated as the number of days between first and last recorded weight. Number of days diet/exercise diaries used. These are raw figures which are adjusted for duration of programme use in multivariate analyses to indicate engagement with the programme. Online forum posts. As forum use was not normally distributed, with the majority of participants not posting any messages and a few posting many times a day, a dichotomised form of this variable was used in all multivariate analyses (any messages posted vs no messages posted). Weight and height. These were self-reported on the website at registration and weight subsequently updated by the user. Height and weights were used to calculate initial and final Body Mass Index (BMI = weight in kg/height in m2). Sex and age. These were routinely recorded on registration with the programme.

### Analysis

Multiple regression and logistic regression were used to compare percentage weight loss and clinically significant weight loss (overweight and obese only) between men and women, adjusting for sex, initial BMI and duration of programme use. All subsequent analyses were carried out separately for men and women. A series of multiple regression models were used to test for factors independently associated with weight loss. In each model the dependent variable was percentage weight loss, and initial BMI, and duration of programme use were included as covariates. Age was not included as a covariate as it was not associated with percentage weight loss in univariate analyses. First, separate analyses were carried out for each of the engagement measures i) number of days on which a food diary had been used, ii) number of days on which an exercise diary had been used, and iii) posting of messages on the online forums (dichotomous). A further multiple regression was carried out including all three measures of engagement, to test the mutually-adjusted associations between each type of engagement and percentage weight loss.

Binary logistic regression was used to establish whether engagement factors were independently associated with clinically significant weight loss (> 5% of initial weight) among users who were overweight or obese. Normal weight participants were excluded from the logistic regression as there are no standards for weight loss conferring clinical benefits in the normal weight population. Independent variables were initial BMI, duration of programme use, and the three engagement variables. All analyses were carried out using SPSS v17.

## Results

Of all service users (n = 8416) subscribing to the website between July 2005 and November 2008, 3621 met the inclusion criteria (women n = 2979, men n = 642), and data from these participants form the basis of the analyses. Those included in the analysis were slightly but significantly older than those not included (36.1 years vs. 35.1 years *t *= 4.2, *p *< .001) and had a higher initial BMI (BMI = 29.2 vs. 28.5 *t *= 5.0, p < .001), but there was no difference in the proportions of men and women (χ^2 ^= .20, ns).

Table 1 shows the characteristics and weight loss of those included in the analysis. Men weighed more than women (99.0 vs 78.0 kg: t = 29.4 *p *< .001) and had a higher initial BMI (31.0.vs 28.9: t = 8.7 *p *< .001). They lost more weight than women (5.6 vs. 3.7 kg: *t *= 6.8, *p *< .001) and lost a higher percentage of their body weight (5.5% vs 4.5% t = 4.3 p < .001). This gender difference in weight loss remained significant in a multiple regression analysis adjusting for initial BMI, and duration of programme use (β = .04, *t *= .39, *p *< .05). Among overweight and obese participants, men were also more likely to achieve a clinically significant weight loss: 47.6% of overweight and obese men lost > 5% of their body weight compared with 40.7% of overweight and obese women (χ^2 ^= 9.3, *p *< .01). A logistic regression showed that the gender difference in clinically significant weight loss was also independent of initial BMI and duration of programme use (Wald χ^2 ^= 7.2, *p *< .01)

**Table 1:** Baseline characteristics and weight change of men and women^1^

	Normal Weight (BMI 18.5- < 25)	Overweight (BMI 25- < 30)	Obese (BMI > 30)	All
Women (n)	816	1150	1015	2979

*Age *	32.3 (8.9)^2^	35.6 (10.3)	37.8 (11.1)	35.5 (10.5)
*Initial BMI *	23.2 (1.3)	27.2 (1.4)	35.3 (4.9)	28.9 (5.8)
*Initial weight (kg)*	63.6 (6.0)	73.5 (6.8)	94.8 (15.2)	78.0 (16.3)
*Weight loss (kg)*	2.1 (2.7)	3.1 (3.8)	5.5 (7.0)	3.7 (5.1)
*Percentage weight loss*	3.2 (4.2)	4.2 (5.1)	5.7(6.6)	4.5 (5.5)
*Clinically significant* *weight loss*^*3 *^*% (n)*	n/a	38.2 (439)	43.5 (442)	40.7 (881)

Men (n)	33	269	340	642

*Age *	35.2 (9.6)	37.9 (9.8)	40.6 (10.2)	39.2 (10.1)
*Initial BMI *	23.5 (1.4)	27.8 (1.4)	34.3 (3.9)	31.0 (4.7)
*Initial weight (kg)*	73.7 (8.7)	88.8 (7.9)	109.6 (15.0)	99.0 (16.9)
*Weight loss (kg)*	2.3 (2.3)	4.2 (4.7)	7.1 (7.6)	5.6 (6.5)
*Percentage weight loss*	3.0 (3.2)	4.7 (5.4)	6.4 (6.4)	5.5 (5.9)
*Clinically significant* *weight loss*^*3 *^*% (n)*	n/a	45.4 (122)	49.4 (168)	47.6 (290)

^1 ^No members had an initial BMI in the underweight range (< 18.5)

^2 ^Values are means (standard deviations) unless otherwise stated.

^3 ^> 5% weight loss: overweight and obese participants only.

### Patterns of website use

Engagement with the website among members varied greatly, with some participants using the website most days and others only accessing it sporadically. The mean number of days that entries were made to food and exercise diaries and the percentage of participants posting on the support forum can be seen in Table 2. Men remained registered with the website for longer than women (187 vs. 170 days respectively; *t *= 2.1, *p *< .05) and made more frequent diet diary entries (56% vs. 52% of registered days; *t *= 2.6, *p *< .05). There was no gender difference in frequency of using the exercise diaries (35% vs. 34% of registered days t = .83, *p *= .17). Almost twice as many women as men posted messages on the online support forums (35% vs. 19% respectively, χ^2 ^= 61.5, *p *< .001).

**Table 2:** Engagement with the Nutracheck website among women and men

	Women (n = 2979)	Men (n = 642)	*p *<^1^
Duration of programme use (days)	169.7 (183.2) 28-1284^2^	186.7 (192.6) 28-1071	.05
Days food diary used	67.0 (74.6) 1-767	76.1 (83.9) 1-938	.01
% of registered days food diary used	52.2 (32.2) 0-100	56.0 (33.5) 0-100	.05
Days exercise diary used	42.3 (55.1) 1-761	45.7 (59.9) 0-770	ns
% of registered days exercise diary used	34.0 (28.5) 0-100	35.0 (27.9) 0-100	ns
Use of online forum (number of messages posted)	26.2 (135.4) 0-3873	6.33 (38.4) 0-537	.001
Any use of online forum % (n)	34.7 (1035)	18.8 (121)	.001

^1^Differences in continuous variables were tested with *t*-test and in categorical variables with χ^2 ^tests.

^2^Mean (standard deviation) range: unless otherwise stated.

### Engagement and weight loss

Hierarchical multiple regression was used to establish whether engagement with the programme independently predicted weight change, after controlling for duration of programme use and initial BMI. In preliminary univariate analyses, age was not significantly associated with percentage weight loss in either men (r = .07, p = .08) or women (r = -.003, p = .86) and so was not included in the regression model. Separate analyses were run for men and women. The results of the multiple regressions can be seen in Table 3. Among women, all engagement measures were significant predictors of percentage weight loss when entered separately into the model. Engagement with food diaries and engagement with exercise diaries accounted for 13% and 9% respectively of the variance in percentage weight loss, while forum use made a small but statistically significant contribution of 2%.

**Table 3:** Website engagement and percentage weight loss: multiple regression analysis

**Independent associations with percentage weight loss**^**1**^				
	Beta	p	Variance explained by engagement R^2 ^change (p)	Variance explained by model Adjusted R^2^
Women				
Food diary engagement	.40	< .001	13% (< .001)	19%
Exercise diary engagement	.33	< .001	9% (< .001)	15%
Forum use (dichotomised)	.14	< .001	2% (< .001)	8%
Men				
Food diary engagement	.49	< .001	18% (< .001)	21%
Exercise diary engagement	.45	< .001	17% (< .001)	20%
Forum use (dichotomised)	.04	.30 ns	0.2% (.29 ns)	3%

**Mutually adjusted associations with percentage weight loss**^**2**^				

	Beta	p	Variance explained by engagement R^2 ^change (p)	Variance explained by model Adjusted R^2^
Women				
Food diary engagement	.36	< .001	13% (< .001)	19%
Exercise diary engagement	.03	.48 ns		
Forum use (dichotomised)	.07	< .01		
Men				
Food diary engagement	.30	< .001	19% (< .001)	22%
Exercise diary engagement	.21	< .01		
Forum use (dichotomised)	-.01	.75 ns		

Women N = 2979 Men N = 642

^1^Covariates: initial BMI, and number of days between first and last recorded weight. Separate analyses for each engagement variable

^2^Covariates as above and all engagement variables included in the model

Among men engagement with food diaries accounted for 18% of the variance in percentage weight loss and engagement with exercise diaries accounted for 17%. Use of the online forums was not a significant predictor of percentage weight loss in men.

When all engagement variables were included in the regression model together, exercise diary adherence became non-significant in women (β = .03, p = .48) and forum use remained non-significant in men. All other engagement variables were significantly associated with percentage weight loss. The engagement variables together accounted for 13% of the variance in percentage weight loss in women and 19% in men.

Logistic regression was used to examine predictors of clinically significant weight loss (defined as loss of > 5% of body weight) among participants who were initially overweight or obese. With initial BMI and duration of programme use in the model, all engagement measures were independently associated with clinically significant weight loss in women (table 4). The odds ratios associated with clinically significant weight loss in the highest tertile of adherence to food and exercise diaries (vs lowest) were 5.1 (p < .001), and 1.5 (p < .05) respectively. The odds ratio for forum use (vs not) was 1.3 (p < .01). Among men there was no significant association between forum use and odds of clinically significant weight loss. The odds ratios associated with clinically significant weight loss in the highest tertile of adherence to food and exercise diaries (vs lowest) were 3.5 (p < .001), and 3.5 (p < .001) respectively.

**Table 4:** Logistic Regression: Factors associated with clinically significant weight loss (> 5%) in a multivariate analysis

	OR (95% CI)	*p *<
**Women**		
Initial BMI	1.03 (1.01-1.04)	.01
Duration of programme use	1.00 (1.00-1.00)	ns
Food diary adherence (tertiles)		
*Low adherence*	1	
*Med adherence*	2.54 (1.91-3.36)	.001
*High adherence*	5.05 (3.51-7.26)	.001
Exercise diary adherence (tertiles)		
*Low adherence*	1	
*Med adherence*	0.80 (0.61-1.04)	ns
*High adherence*	1.46 (1.03-2.04)	.05
Use of forums		
*Non-user*	1	
*Forum user*	1.33 (1.09-1.63)	.01

**Men**		
Initial BMI	1.04 (1.01-1.09)	.05
Duration of programme use	1.00 (1.00-1.00)	ns
Food diary adherence (tertiles)		
*Low adherence*	1	
*Med adherence*	1.49 (0.87-2.54)	.ns
*High adherence*	3.45 (1.78-6.80)	.001
Exercise diary adherence (tertiles)		
*Low adherence*	1	
*Med adherence*	1.52 (0.91-2.53)	ns
*High adherence*	3.48 (2.03-7.49)	.001
Use of forums		
*Non-user*	1	
*Forum user*	0.83 (0.52-1.33)	ns

## Conclusions

As is commonly the case with groups of people seeking to lose weight, the sample was disproportionately female, with more than four times as many women as men. There were some differences in how men and women used the programme; notably women were more likely than men to use the online support forum, in keeping with the finding that women are more likely to use Internet communication for social support than men in relation to a variety of health issues [30,34]. Men using the programme lost a higher percentage of their body weight than women (5.5% vs 4.5%), an effect which was independent of initial BMI and duration of website use. Gender differences in weight loss are a common observation in weight loss programmes, and both motivational and biological factors have been suggested as explanations. Men who are trying to lose weight often have a higher BMI, fewer prior weight loss attempts then women, and greater weight loss self-efficacy [31,35]. They are also more likely than women to attempt to lose weight in response to a medical event or other health trigger; a factor which is associated with greater weight loss success in both men and women [36].

Some of the participants meeting the inclusion criteria for this study made only limited use of the programme, although all those included in the analyses registered a weight on at least two occasions at least 28 days apart. Greater engagement, in terms of frequency of diet and exercise diary use and message posting on the online forums, was associated with weight loss, suggesting that self-monitoring and social support were mechanisms through which the programme had its effect. The independent effects associated with using the online forum were small, and only seen in women, but the weight loss associated with greater food diary use was more substantial and was consistent between men and women. Although both men and women made similar use of the exercise diary facility, adherence to the exercise diary was more strongly associated with weight loss for men than women. This is in keeping with evidence that weight loss through exercise may be more effective for men than for women [37,38].

It has been suggested that self monitoring operates by enhancing attentional focus on the desired behaviour [19,39,40]. The results presented here support this view, suggesting that the frequency of performing simple diet and exercise monitoring behaviour can facilitate weight loss without reference to the content of the diaries. Use of the peer-support forums was less widespread than use of the diaries, although the number of users who read the forums without posting may be much higher. Other studies have found that commercial programmes with large numbers of members offer good opportunities for peer support, and that use of such facilities is modestly associated with weight loss [41]. A decision to post messages on the forum may be prompted by a variety of different circumstances. Users can use the forums to post questions, provide information and advice to others, or seek emotional and motivational support. However, in all cases it seems likely that message posting reflects commitment to and engagement with the programme, and as such may be a marker for level of motivation. Future research should address how far forum use *per se *promotes or facilitates weight loss success, or whether self-selection accounts for the association between weight loss and use of online support.

The retrospective nature of this study is a limitation; however, many people seeking to lose weight do so using largely self-guided approaches and the study of engagement and outcomes in these groups may not always be compatible with the additional intervention inherent in prospective studies, or the context of a trial [16]. The effects of paying for a weight loss service (as was the case for participants in this analysis) is not clear. Previous studies of commercial programmes, which have often been in the form of RCTs, have generally involved the payment of subscription fees by the study organisers [6,41], and it is plausible that paying for a weight loss service might affect motivation and weight loss outcomes.

This study demonstrates that engagement with self monitoring and support tools in a self-guided, direct-to-consumer, online environment is associated with weight control success.

## Competing interests

Jane Wardle and Fiona Johnson declare they have no competing interest to disclose. The preparation of this paper was assisted by Rachel Hartley and Tim Vryenhoef of NutraTech Ltd, the parent company of the Nutracheck.co.uk online service. TV prepared the requested data for analysis and commented on the analysis. RH provided details of the Nutracheck energy requirement calculations and commented on the draft article.

## Authors' contributions

FJ planned the analysis strategy, analyzed the data and drafted the article in collaboration at all stages with JW. Both authors read and approved the final manuscript.
